# Acute Aβ12‐28P administration targeting apoE/Aβ interaction rescues synaptic function and cognitive decline in advanced‐stage Alzheimer’s disease

**DOI:** 10.1002/alz.093081

**Published:** 2025-01-09

**Authors:** Shuo Chen, Nikolas Lam, Zhiheng Li, Ding Wang, Pengfu Liu, Victoria Berbano, Yu Xie, Tyra Nguyen, Yvonne Xu, John Yang, Karen Ha, Leonardo Semana, Rachel Dias, Arjun V. Masurkar, Thomas Wisniewski

**Affiliations:** ^1^ NYU Grossman School of Medicine, New York, NY USA; ^2^ New York University, New York, NY USA

## Abstract

**Background:**

Apolipoprotein E4 (apoE4) has been identified as the major genetic risk factor for late onset Alzheimer’s disease (AD). Our lab has demonstrated that chronic administration of Aβ12‐28P, a synthetic peptide that blocks apoE4/Aβ binding, in middle‐aged transgenic AD mice significantly ameliorates pathology progression, resulting in reduced Aβ plaques deposition and cerebral amyloid angiopathy (CAA) along with improved memory and cognition. However, whether blocking apoE4/Aβ interaction by Aβ12‐28P also has an ameliorating effect on the neuronal and cognitive function of old AD mice where Aβ pathology has been extensively developed remains unknown.

**Method:**

Geriatric SwDI/APOE4 mice (18‐24 months old) were intraperitoneally injected daily with Aβ12‐28P, while control mice received only saline. Behavioral tests including anxiety in open field, locomotor activity, T‐maze, object recognition, and radial arm maze were performed 2 hours post‐injection to assess the acute effect of Aβ12‐28P. After 3 weeks of behavioral testing with daily treatment, acute hippocampal slices were prepared and subjected to *in vitro* physiology experiments to evaluate long‐term potentiation (LTP) of Schaffer collateral input to CA1. On the recording day Aβ12‐28P or saline was injected 2 hours before slice preparation.

**Result:**

Acute Aβ12‐28P treatment resulted in significant cognitive improvement. While the treated mice did not show differences in locomotor activity, significantly enhanced cognitive performances were observed, including improved performance in one‐day T‐maze test (*n* = 10/group, p = 0.0005), one‐day novel object recognition test (*n* = 6/group, p = 0.018), and multiple‐days radial arm maze test (*n* = 8‐9/group, p = 0.0008). Aβ12‐28P administration also significantly reduced anxiety in open field (*n* = 6/group, p = 0.0081). We further compared the hippocampal Schaffer collateral LTP in slices from Aβ12‐28P‐treated versus non‐treated mice. Slices from the treated animals showed significantly enhanced LTP (*n* = 9‐10/group, p < 0.0001), suggesting rescued synaptic plasticity.

**Conclusion:**

These results suggest that acute Aβ12‐28P administration blocking apoE/Aβ binding rescues synaptic plasticity and cognitive decline in old AD mice where extensive Aβ pathology has been developed. Our finding opens the possibilities to apply the strategy of targeting apoE/Aβ interaction for alleviating advanced‐stage AD symptoms in addition to ameliorating Aβ deposition during AD progression.

